# The p.V37I Exclusive Genotype Of *GJB2*: A Genetic Risk-Indicator of Postnatal Permanent Childhood Hearing Impairment

**DOI:** 10.1371/journal.pone.0036621

**Published:** 2012-05-04

**Authors:** Lei Li, Jingrong Lu, Zheng Tao, Qi Huang, Yongchuan Chai, Xiaohua Li, Zhiwu Huang, Yun Li, Mingliang Xiang, Jun Yang, Guoyin Yao, Yu Wang, Tao Yang, Hao Wu

**Affiliations:** 1 Department of Otolaryngology–Head and Neck Surgery, Xinhua Hospital, Shanghai Jiaotong University School of Medicine, Shanghai, China; 2 Ear Institute, Shanghai Jiaotong University, Shanghai, China; 3 Shanghai Children's Medical Center, Shanghai, China; 4 Shanghai Child Health Care Institute, Shanghai, China; Stanford University School of Medicine, United States of America

## Abstract

Postnatal permanent childhood hearing impairment (PCHI) is frequent (0.25%–0.99%) and difficult to detect in the early stage, which may impede the speech, language and cognitive development of affected children. Genetic tests of common variants associated with postnatal PCHI in newborns may provide an efficient way to identify those at risk. In this study, we detected a strong association of the p.V37I exclusive genotype of *GJB2* with postnatal PCHI in Chinese Hans (*P* = 1.4×10^−10^; OR 62.92, 95% CI 21.27–186.12). This common genotype in Eastern Asians was present in a substantial percentage (20%) of postnatal PCHI subjects, and its prevalence was significantly increased in normal-hearing newborns who failed at least one newborn hearing screen. Our results indicated that the p.V37I exclusive genotype of *GJB2* may cause subclinical hearing impairment at birth and increases risk for postnatal PCHI. Genetic testing of *GJB2* in East Asian newborns will facilitate prompt detection and intervention of postnatal PCHI.

## Introduction

Hearing is critical for normal development and acquisition of speech. Early detection with intervention and habilitation for permanent childhood hearing impairment (PCHI) leads to significantly better speech, language and cognitive development [1], [2], [3]. While universal newborn hearing screening (NHS) has been successful in detection of hearing impairment at birth, early detection of postnatal PCHI, which occurs at an estimated prevalence of 0.25‰–0.99‰ in children aged 9 years [4], [5], remains a challenge [6]. A series of risk factors, featuring the clinical, family or medical history, has been recommended by the Joint Committee on Infant Hearing to prompt continued audiological monitoring of those at higher risk of postnatal PCHI [7]. These risk factors, however, were not present in a significant percentage (52%–67.5%) of children with postnatal PCHI in several previous reports [5], [8].

Genetic testing in newborns has been proposed as a potentially effective way to detect postnatal PCHI that are either not present at birth or associated with subclinical hearing loss [6]. This practice, however, is hindered by limited knowledge of common genetic variants associated with postnatal PCHI. Mutations in the *GJB2* gene are the most common genetic cause for deafness [9], [10], [11]. Though most cases of *GJB2*-associated hearing impairment are congenital, a small number of individuals with bi-allelic *GJB2* mutations have been reported who initially passed the NHS but were subsequently identified with hearing loss in their infancy or early childhood [12], [13]. No *GJB2* mutation, however, has been reported specifically associated with postnatal PCHI.

The p.V37I mutation of *GJB2* is highly prevalent in East Asians, with a allele frequency of 4.3% in Thai [14], 1.0% in Japanese [15], 0.6% in Korean [16], and 6.2% in Chinese (determined in this study). When present in homozygosity or in compound heterozygosity with another truncating mutation of *GJB2* (referred as “the exclusive p.V37I genotype” because only the p.V37I allele(s) have the potential to produce protein product), it can lead to non-fully penetrant, mild-to-moderate hearing impairment that is sometimes progressive [17], [18]. In this study, we hypothesized that the p.V37I exclusive genotype of *GJB2* is associated with postnatal PCHI and serves as a genetic risk-indicator for PCHI.

## Methods

### Subjects and ethics statement

A total of 45 unrelated Chinese Han children with bilateral postnatal PCHI (mean age of 7 years, 95% CI 3.9–10.1 years) were recruited from Shanghai, China. Among them, fourteen children (group 1) passed the NHS in both ears but developed bilateral postnatal PCHI (mean hearing threshold 51.3 dB nHL, 95% CI 37.5–65.1 dB nHL) before age 9 years. Ten of these 14 children were identified and recruited though a large scale preschool hearing screen conducted by the Xinhua Hospital and the Shanghai Child Health Care Institute during year 2009–2010, and the remaining four through their clinic visits to the Xinhua Hospital. Thirty-one children (group 2) did not receive NHS at birth but passed the pediatric hearing tests of their kindergarten enrollment physical examination at the age of 2–4 years. All these 31 children developed bilateral PCHI (mean hearing threshold 50.6 dB nHL, 95% CI 40.8–60.4 dB nHL) before age 9 years and were recruited clinically through Xinhua Hospital.

**Table 1:** Genotyping results of *GJB2* in control and subject groups.

Catagory	Total	−/−	p.V37I/−	c.235delC/−	T */−	p.V37I/p.V37I	p.V37I/c.235delC
Control newborns	1516	1303	175	22	10	5	1
Postnatal PCHI group 1	14	7	0	2	2	3	0
Postnatal PCHI group 2	31	22	3	0	0	4	2
Newborns passed the initial hearing screen	1405	1213	160	21	9	1	1
Newborns passed the repeat hearing screen	99	83	12	1	1	2	0
Newborns passed the referral diagnosis	173	132	21	8	2	8	2

* T: Other truncating *GJB2* mutations identified in this study including c.299_300delAT, c.176del16 and c.507insAACG.

A total of 1516 Chinese Han newborns were recruited from Putuo Women and Children Hospital, Shanghai during 2009. Follow-up of their NHS results showed that 1405 newborns passed the initial hearing screen, 99 newborns failed the initial screen but passed the repeat screen, and 12 newborns failed both screens (Hearing diagnostic results unknown). We also recruited 173 Chinese Han infants who failed both NHSs but were subsequently diagnosed as normal hearing (hearing threshold <35 dB nHL in both ears and passed all comprehensive auditory evaluation in the referral hearing diagnosis conducted within six months of age) from the Shanghai Children's Medical Center, one of the major Children Hearing Impairment Diagnosis Centers in Shanghai.

All subjects or their parents gave written, informed consent to participate in this study. This study was approved by the Ethics Committee of the Shanghai Jiaotong University School of Medicine, Xinhua Hospital and was in compliance with the Declaration of Helsinki.

### Hearing screens and audiological evaluation

The newborn hearing screening of the subjects in this study were preformed uniformly through the Universal Newborn Hearing Screening Program (UNHSP) in Shanghai following standardized testing criteria and procedures as previously decribed [19]. This city-wide screening program was promoted and legislated by the municipal government and was performed as a hospital based, two-stage screening procedure since 2002. Newborns were initially screened on Day 3 post-birth by the transient-evoked otoacoustic emission (TEOAE), and those testing positive underwent a repeat outpatient screening on Day 42. Infants failed both screens were referred for audiological evaluation at one of the Children Hearing Impairment Diagnosis Centers in Shanghai. Diagnostic process for hearing loss in newborns included otoscope examination, tympanometry, automated auditory brainstem response (AABR), distortion products otoacoustic emission (DPOAE), auditory steady-state response (ASSR) and behavior hearing test.

Hearing tests of the pre-kindergarten children in Shanghai were routinely performed during their kindergarten enrollment physical examination by the Shanghai Child Health Care Institute using pediatric audiometers. In this study, a large scale preschool hearing screens were also performed in 21427 children between age 3 and 6 years in Shanghai by the Xinhua Hospital and the Shanghai Child Health Care Institute between October 2009 and September 2010 as previously described [20]. Pediatric audiometry testing was uniformly carried out using the DK-5610 Assens (Interacoustics A/S, Denmark) device. Each child was tested under frequencies of 1, 2, and 4 kHz at 20 dB HL for three times. A pass result was given if the child's responses were judged to be clinically reliable for at least two times for each frequency. This pediatric audiometry testing was deemed sensitive as preschool children with mild hearing impairment (25–40 dB HL) can be consistently detected using this method in our previous study [20]. Those who failed the initial preschool screen were re-tested one month later. Children who failed both tests were referred to Xinhua Hospital for detailed audiological evaluation.

Audiological evaluations were performed for all subjects with postnatal PCHI, including otoscope examination, tympanometry, pure tone audiometry (PTA), play PTA, visual reinforcement audiometry and auditory brainstem response (ABR). Children with conductive hearing impairment due to otitis media with effusion (OME) and eustachian tube dysfunction were excluded for further study. Mean hearing threshold was calculated by pure tone averages in the better ear at 0.5, 1, 2 and 4 kHz. Bilateral permanent childhood hearing impairment (PCHI) was defined as mean hearing threshold exceeded 40 dB HL [4], [5].

### Genetic and statistical analysis

Genetic and statistical analyses of the study were performed in the Molecular Biology of Hearing and Deafness Research Laboratory, Xinhua Hospital. Genomic DNA was extracted from either whole blood samples or fresh buccal swab samples. Mutation screening of the *GJB2* gene was performed by PCR amplification and bidirectional sequencing as previously described [21]. The p.V37I exclusive genotype of *GJB2* was defined as either homozygosity of p.V37I or compound heterozygosity of p.V37I and another *GJB2* truncating mutation. Fisher's exact test and the odds ratio calculation were used to compare the prevalence of the p.V37I exclusive genotype of *GJB2* among different subject groups. *P* values were presented as the result of two-tailed analysis.

## Results

### Association of the *GJB2* p.V37I exclusive genotype with postnatal PCHI

Mutation screening of *GJB2* was performed in 45 Chinese Han children with postnatal PCHI and 1516 ethnically-matched control newborns (Table 1). From controls, we determined the allele frequency of the p.V37I mutation as 6.2% (187/3032), followed by that of c.235delC as 0.8% (23/3032). Consistent with this result, homozygosity of p.V37I (5 in 1516 newborns) and compound heterozygosity of p.V37I/c.235delC (1 in 1516 newborns) were the only two bi-allelic mutant genotypes of *GJB2* observed in this study (termed as the p.V37I exclusive genotype as p.V37I is the only non-truncating allele of *GJB2*).

The prevalence of the p.V37I exclusive genotype of *GJB2* was 21.4% (3/14) in postnatal PCHI group 1 who passed NHS at birth, and 19.4% (6/31) in postnatal PCHI group 2 who passed the pediatric hearing tests during their kindergarten enrollment physical examination, both significantly higher than the prevalence of 0.4% (6/1516) in the ethnically matched control newborns (*P* = 9.0×10^−5^, OR = 68.63, 95% CI 5.20–309.93 for group1; *P* = 9.8×10^−8^, OR = 60.40, 95% CI 18.22–200.27 for group 2; *P* = 1.4×10^−10^, OR = 62.92, 95% CI 21.27–186.12 for the combined postnatal PCHI group, Table 2). These results showed that the p.V37I exclusive genotype of *GJB2* is strongly associated with postnatal PCHI and is present in a substantial percentage (20.0%, 9/45) of postnatal PCHI cases in Chinese.

**Table 2:** Prevalence of the p.V37I exclusive genotype in control newborns and children with postnatal PCHI.

	Total	Exclusive p.V37I genotype (%)	*p*- value*
Control newborns	1516	6 (0.40)	-
Postnatal PCHI group 1	14	3 (21.4)	9.0×10^−5^
Postnatal PCHI group 2	31	6 (19.4)	9.8×10^−8^
Combined postnatal PCHI group	45	9 (20.0)	1.4×10^−10^

* Calculated by Fisher's exact test, two tailed analysis in comparison to control newborns.

### Increased prevalence of the p.V37I exclusive genotype in newborns with subclinical hearing loss

The 1516 control newborns can be categorized into three sub-groups based on their NHS results: 1405 newborns who passed the initial screen (sub-group 1), 99 newborns who failed the initial screen but passed the repeat screen (sub-group 2), and 12 newborns who failed both screens (sub-group 3). The prevalence of the p.V37I exclusive genotype was 2.0% (2/99) in sub-group 2, significantly higher than the prevalence of 0.14% (2/1405) in sub-group 1 (*P* = 0.024, Table 3). Because of the small sample size and the unavailability of the hearing diagnostic results of sub-group 3, we separately recruited 173 newborns that failed both NHS tests but were diagnosed with normal hearing on their referral evaluations. The prevalence of the p.V37I exclusive genotype was 5.8% (10/173) in this group, further higher than that in sub-group 1 (*P* = 1.7×10^−8^). The increased prevalence of the p.V37I exclusive genotype in normal-hearing newborns who failed at least one NHS suggested that the p.V37I exclusive genotype causes subclinical or borderline slight hearing loss at birth and increases risk for postnatal PCHI.

**Table 3:** Prevalence of the p.V37I exclusive genotype in normal hearing newborns with different NHS results.

	Total	Exclusive p.V37I genotype (%)	*p*- value*
Newborns who passed the initial hearing screen	1405	2 (0.14)	-
Newborns who passed the repeat hearing screen	99	2 (2.0)	0.024
Newborns who passed the referral evaluation	173	10 (5.8)	1.7×10^−8^

* Calculated by Fisher's exact test, two tailed analysis in comparison to newborns who passed the initial hearing screen.

## Discussion

Our study identified the p.V37I exclusive genotype of *GJB2* as a common genetic risk-indicator of postnatal PCHI in Chinese Han population. This genotype has been shown associated with mild-to-moderate, sometimes progressive hearing impairment in patients with various ages. Protein expression study also confirmed that unlike other truncating mutations of *GJB2* that are associated with severer hearing impairment and lead to abnormal subcellular localization of the mutant protein, the p.V37I mutant protein has normal membrane expression as the wild type protein, suggesting its retaining of at least part of the protein function [22]. The clinical phenotype of the p.V37I mutation fits its potential role in postnatal PCHI well as most late-onset sensorineural hearing impairment is progressive and is not as severe as the congenital hearing impairment. In this study, we found that the p.V37I exclusive genotype is strongly associated with postnatal PCHI (*P* = 1.4×10^−10^) and is present in a substantial percentage (20.0%) of children with postnatal PCHI (Table 2). Homozygosity of p.V37I is the primary p.V37I exclusive genotype, which was present in 3.3‰ (5/1516) of control newborns and 15.6% (7/45) of children with postnatal PCHI (Table 1). Although p.V37I/c.235delC, which was present in 0.66‰ (1/1516) of control newborns and 4.4% (2/45) of children with postnatal PCHI, is the only other biallelic mutant genotype of *GJB2* observed in our study, compound heterozygous mutations of p.V37I and one other truncating mutation of *GJB2* should also be considered as a genetic risk for postnatal PCHI due to their similar molecular pathogenesis mechanism.

Among newborns with the p.V37I exclusive genotype, it remains unclear what percentage will develop PCHI congenitally and what percentage will develop PCHI postnatally. However, our study showed that even in those who were “cleared” during NHS or referral hearing diagnosis (In the UNHS program in China, referred newborns with hearing threshold lower than 35 dB nHL for both ears were usually cleared as normal hearing without further intervention.), the p.V37I exclusive genotype may still cause subclinical or borderline slight hearing loss at birth and increase the risk for postnatal PCHI, as prevalence of the p.V37I exclusive genotype increased significantly in normal-hearing newborns who failed at least one NHS (Table 3). Therefore newborns with the p.V37I exclusive genotype represent a genetically distinct group that is predisposed to mild, sometimes subclinical hearing loss at birth, and this hearing loss may progress and become apparent at later stage.

Based on these results, we propose that wide-spread genetic testing for the *GJB2* p.V37I exclusive genotype is warranted in East Asian newborns. This screening can be performed either universally in all newborns or preferentially in those who fail at least one NHS. One of the interesting findings in this study is the increased frequency of the p.V37I exclusive genotype in newborn who failed at least one NHS compared to those who passed the initial NHS. It will be important to study whether the former newborns have a higher likelihood to develop postnatal PCHI. If this turns out to be the case, then different strategies for incorporating genetic testing into NHS should be evaluated to identify which strategy would be optimal.

In summary, our study revealed a strong association of PCHI with the p.V37I exclusive genotype of *GJB2*. We also found that the prevalence of this genotype increased significantly in normal-hearing newborns who failed at least one newborn hearing screen. Based on these results, we concluded that the p.V37I exclusive genotype of *GJB2* causes subclinical hearing loss at birth and increases risk for postnatal PCHI. Our study may form basis to support wide-spread genetic test of the p.V37I exclusive genotype of *GJB2* in East Asian newborns. Continued audiologic monitoring should be offered to children who carry this genetic variant to facilitate prompt detection and intervention of postnatal PCHI, which in turn will benefit the speech, language and cognitive development of the affected children.
